# Helminth Coinfection Is Associated With Enhanced Plasma Levels of Matrix Metalloproteinases and Tissue Inhibitor of Metalloproteinases in Tuberculous Lymphadenitis

**DOI:** 10.3389/fcimb.2021.680665

**Published:** 2021-07-19

**Authors:** Gokul Raj Kathamuthu, Kadar Moideen, Kannan Thiruvengadam, Rathinam Sridhar, Dhanaraj Baskaran, Subash Babu

**Affiliations:** ^1^ National Institutes of Health—NIRT—International Center for Excellence in Research, Chennai, India; ^2^ National Institute for Research in Tuberculosis (NIRT), Chennai, India; ^3^ Government Stanley Medical Hospital, Chennai, India; ^4^ Laboratory of Parasitic Diseases, National Institute of Allergy and Infectious Diseases, National Institutes of Health, Bethesda, MD, United States

**Keywords:** extra-pulmonary TB, helminth infection, matrix metalloproteinases, tissue inhibitors of metalloproteinases, Luminex ELISA

## Abstract

Matrix metalloproteinases (MMPs) are crucial for tissue remodeling and repair and are expressed in diverse infections, whereas tissue inhibitors of metalloproteinases (TIMPs) are endogenous inhibitors of MMPs. However, the interaction of MMPs and TIMPs in tuberculous lymphadenitis (TBL), an extra-pulmonary form of tuberculosis (EPTB) and helminth (Hel+) coinfection is not known. Therefore, this present study investigates the levels of circulating MMPs (1, 2, 3, 7, 8, 9, 12, 13) and TIMPs (1, 2, 3, 4) in TBL individuals with helminth (*Strongyloides stercoralis* [Ss], hereafter Hel+) coinfection and without helminth coinfection (hereafter, Hel-). In addition, we have also carried out the regression analysis and calculated the MMP/TIMP ratios between the two study groups. We describe that the circulating levels of MMPs (except MMP-8 and MMP-12) were elevated in TBL-Hel+ coinfected individuals compared to TBL-Hel- individuals. Similarly, the systemic levels of TIMPs (1, 2, 3, 4) were increased in TBL-Hel+ compared to TBL-Hel- groups indicating that it is a feature of helminth coinfection *per se*. Finally, our multivariate analysis data also revealed that the changes in MMPs and TIMPs were independent of age, sex, and culture status between TBL-Hel+ and TBL-Hel- individuals. We show that the MMP-2 ratio with all TIMPs were significantly associated with TBL-helminth coinfection. Thus, our results describe how helminth infection has a profound effect on the pathogenesis of TBL and that both MMPs and TIMPs could dampen the immunity against the TBL-Hel+ coinfected individuals.

## Introduction

Tuberculosis (TB) poses a significant public threat and kills nearly 1.2 million individuals globally, with 90% of the infection emerging in developing nations (Sanou et al., 2015; WHO, 2019). Both cellular and humoral immunity are necessary to control *Mycobacterium tuberculosis* (Mtb) infection. They often require activation of monocytes, macrophages, T cells (particularly IFNγ), B cells, natural killer (NK) cells, and dendritic cells (DC) (Flynn and Chan, 2001; Saunders and Britton, 2007; O’Garra et al., 2013). However, during adverse circumstances, Mtb escapes from the protective immune environment or granuloma and thereby advances to different forms of TB such as active pulmonary TB (ATB) and extrapulmonary TB (Russell et al., 2009; O’Garra et al., 2013). Similarly, helminth parasites are well-known multifaceted eukaryotic organisms that cause chronic illness in humans. Strongyloidiasis caused by *Strongyloides stercoralis* affects about 50-100 million persons worldwide and they exhibit a free-living auto infective cycles followed by prolonged infection (Babu and Nutman, 2013; Puthiyakunnon et al., 2014; Toledo et al., 2015). They are the potential regulators of defensive immunity against different forms of TB (Metenou et al., 2012). Helminth infection usually appears in resource limited settings and accounts for a massive degree of geographical overlap with TB disease (Salgame et al., 2013) and both together cause infection (Allen and Maizels, 2011; O’Garra et al., 2013).

Previous epidemiological and experimental reports have revealed that both systemic and intestinal helminths negatively influence TB disease (Salgame et al., 2013). In addition, both local and systemic inflammatory responses and innate parameters also impact the severity and pathogenesis of TB disease. Most importantly, certain non-specific inflammatory markers like matrix metalloproteinases (MMPs) and acute phase proteins are employed to measure the severity of TB disease and are considered to be an indispensable element (Walzl et al., 2011; Wallis et al., 2013). These MMPs belong to a broad family of zinc- and calcium-dependent proteolytic enzymes. Different studies highlighted the critical role of MMPs in inflammatory cell migration, cellular recruitment, tissue remodeling, destruction of matrix and non-matrix proteins, disease pathogenesis, and immune response alteration (Sundararajan et al., 2012; Bruschi and Pinto, 2013; Ugarte-Gil et al., 2013). MMP activity is regulated by endogenous inhibitors called tissue inhibitors of metalloproteinases (TIMPs). TIMPs are also extremely crucial for the remodeling and repair of normal and pathological tissues following destruction induced by MMPs (Sundararajan et al., 2012; Ugarte-Gil et al., 2013). In pulmonary TB (PTB), MMPs are majorly important in cavitary lung disease and aid in dissemination (Elkington et al., 2011a). Earlier studies have displayed that circulating levels of MMPs were reduced in active TB-helminth coinfected individuals than those with active TB alone (George et al., 2014). Similarly, increased systemic levels of MMPs and TIMPs are the characteristic feature of filarial infection (Anuradha et al., 2012). However, to the best of our knowledge, no studies have reported the circulating levels of MMPs and TIMPs in TBL helminth coinfection. Hence, we examined the same in this study and show that TBL-Hel+ coinfected individuals are associated with enhanced plasma levels of MMPs and TIMPs compared to TBL-Hel- individuals.

## Methods

### Ethics Statement

The study was sanctioned and approved by the National Institute for Research in Tuberculosis (NIRT)-Institutional Review Board (NIRTIEC2010007), India, and informed written consent was obtained from all the study participants.

### Study Subjects

We recruited a group of 88 study participants with TBL disease, among them one group of individuals were infected with Ss infection (n=44, Hel+) and the other group of individuals had only TBL (without Ss infection [Hel-]). The study individuals were well defined and have been reported previously (Kathamuthu et al., 2019). We have utilized the same set of samples in this experiment and their detailed demographics and hematological profile were reported (**Tables 1**, **2**). TBL individuals were diagnosed based on either histopathology or bacteriological culture using infected lymph node samples. The disease severity was calculated using the homogenized lymph node cultures and calculated based on their grades [0 (no colonies)/1+ (20–100 colonies)/2+(>100 colonies)], which were determined by the growth of Mtb on Lowenstein-Jensen solid media (Mitchison and Aber, 1974). The coinfected individuals had higher severity than the non-coinfected individuals (**Table 1**). Helminth (Ss) infection was detected by the existence of IgG antibodies to the 31-kDa recombinant NIE antigen (Bisoffi et al., 2014; Buonfrate et al., 2015). The presence of Ss infection was confirmed only using ELISA and not by stool microscopy investigation. The study individuals were anti-tuberculosis and anthelmintic treatment naïve and not affected with any disseminated strongyloidiasis infection. They were all BCG vaccinated and reported negative for filarial infection (based on TropBio ELISA results) and human immune deficiency virus (HIV) infection.

**Table 1:** Demographics of the study population.

Study Demographics	TBL-Hel+	TBL-Hel-	P Value
Number of subjects recruited (n)	44	44	NS
Gender (M/F)	12/32	14/30	NS
Median age in years (range)	23 (18-53)	25 (19-59)	NS
Culture grade (0/1+/2+)	7/30/7	15/28/1	0.028 ^a^
NIE IgG antibody titres	484.77 (291-1202)	174.3 (38-281.75)	<0.0001^b^

^a^Calculated using the Chi-square test; NS, Not significant. ^b^Calculated using the Mann-Whitney test.

**Table 2:** Hematological profile of the study population.

Hematological profile	TBL-Hel+	TBL-Hel-	P value^a^
Whole blood cells (10^3^/litre)	6.01 (4.9-10.9)	6.36 (3.80-11.9)	NS
Red blood cells (10^6^/litre)	3.98 (3.94-8.9)	4.14 (3.92-5.44)	NS
Lymphocytes (%)	24.53 (14.2-48.1)	28.37 (17.6-43.4)	**0.0497**
Monocytes (%)	5.20 (2.0-11.7)	7.08 (2.9-20.8)	**0.0207**
Eosinophils (%)	3.02 (1.6-8.7)	1.70 (1.0-3.2)	**0.0316**
Basophils (%)	0.85 (0.3-6.7)	0.85 (0.2-2.2)	NS
Neutrophils (%)	50.65 (42.9-76.9)	49.77 (45.8-71.6)	**0.0287**
Platelets (10^3^/litre)	262.13 (152-482)	273.04 (179-654)	NS
Hemoglobin (g/dl)	9.7 (8.0-18.3)	11.02 (8.0-14.8)	NS

a Calculated using the Mann-Whitney test; NS, Not significant. Bold values indicates the significance.

### Plasma Separation

10 ml of peripheral blood samples were collected in sodium heparin tubes and centrifuged at 2600 revolutions per minute (rpm) for 10 minutes at 4°C. Then the plasma was meticulously transferred in a fresh screw cap vial and stored at -80°C for further use.

### Luminex ELISA

The circulating levels of MMP-1, MMP-2, MMP-3, MMP-7, MMP-8, MMP-9, MMP-12, MMP-13 (catalogue number FCSTM07-8), and human TIMP-1, TIMP-2, TIMP-3 and TIMP-4 (catalogue number LKTM003) were measured using a Luminex kit (R&D Systems). The experiments were performed using Bio-Plex^®^ MAGPIX™ multiplex reader (BIO-RAD) and the data were exported using Bio-plex manager 6.1 version.

### Statistical Analysis

All the data were analyzed using GraphPad Prism version 8.0 (GraphPad Software Inc., San Diego, CA). Geometric means (GM) were used to measure the central tendency and statistically significant differences between the study groups were analyzed using non-parametric Mann-Whitney U test. Univariate and multivariate (regression) analyses were performed using STATA/MP version 16.0.

## Results

### TBL-Hel+ Individuals Exhibit Significantly Heightened Circulating Levels of MMPs

**Figure 1:**
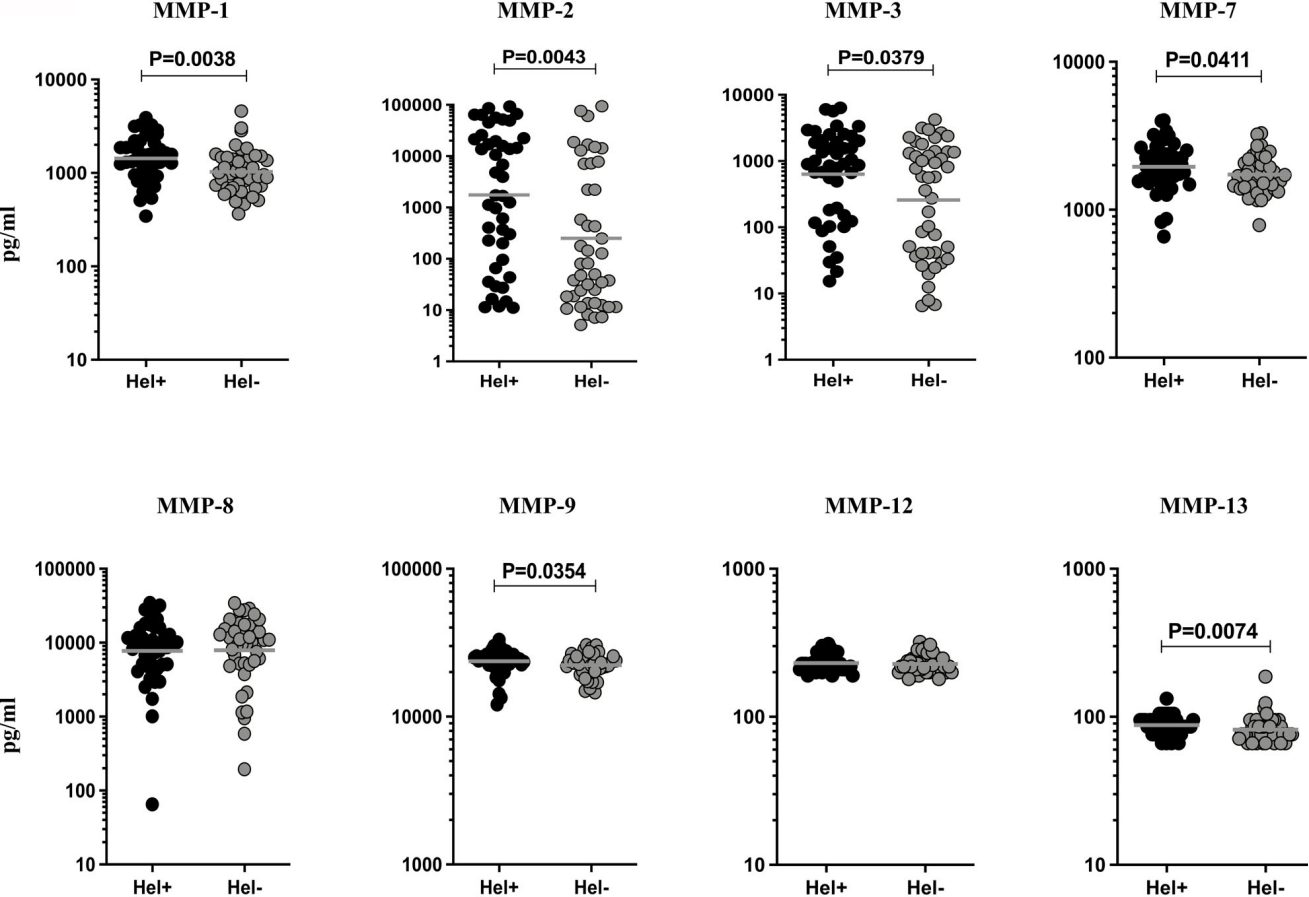
Circulating levels of matrix metalloproteinases (MMPs) were elevated in TBL-Hel+ coinfected individuals. The systemic levels of MMPs (1, 2, 3, 7, 8, 9, 12, 13) were examined in TBL-Hel+ (n = 44) and TBL-Hel- (n = 44) individuals. The results were reported as scatter plots with each circle denoting a single individual. The bar indicates the geometric mean and P values (p < 0.05, *p < 0.01**p < 0.001***p < 0.0001****) were calculated using Mann-Whitney U test.

To measure the impact of helminth coinfection on TBL disease, we analysed the circulating levels of MMPs (1, 2, 3, 7, 8, 9, 12, 13) in TBL-Hel+ compared to TBL-Hel- individuals (**Figure 1**). As shown in **Figure 1**, the circulating levels of MMP-1 (geometric mean (GM) of Hel+ is 1433 pg/ml *vs* 1026 pg/ml in Hel-, p=0.0038), MMP-2 (GM of Hel+ is 1750 pg/ml *vs* 250.8 pg/ml in Hel-, p=0.0043), MMP-3 (GM of Hel+ is 632.2 pg/ml *vs* 257.5 pg/ml in Hel-, p=0.0379), MMP-7 (Hel+ is 1954 pg/ml *vs* 1727 pg/ml in Hel-, p=0.0411), MMP-9 (GM of Hel+ is 23691 pg/ml *vs* 22336 pg/ml in Hel-, p=0.0354) and MMP-13 (Hel+ is 87.75 pg/ml *vs* 81.81 pg/ml in Hel-, p=0.0074) were significantly elevated in in TBL-Hel+ compared to TBL-Hel- individuals. In contrast, the plasma levels of MMP-8 (Hel+ is 7775 pg/ml *vs* 7912 pg/ml in Hel-) and MMP-12 (Hel+ is 231 pg/ml *vs* 228.1 pg/ml in Hel-) were not significantly different between TBL-Hel+ compared to TBL-Hel- individuals. Therefore, TBL-Hel+ coinfection is associated with increased circulating levels of MMPs.

### TBL-Hel+ Individuals Exhibit Significantly Heightened Systemic Levels of TIMPs

**Figure 2:**
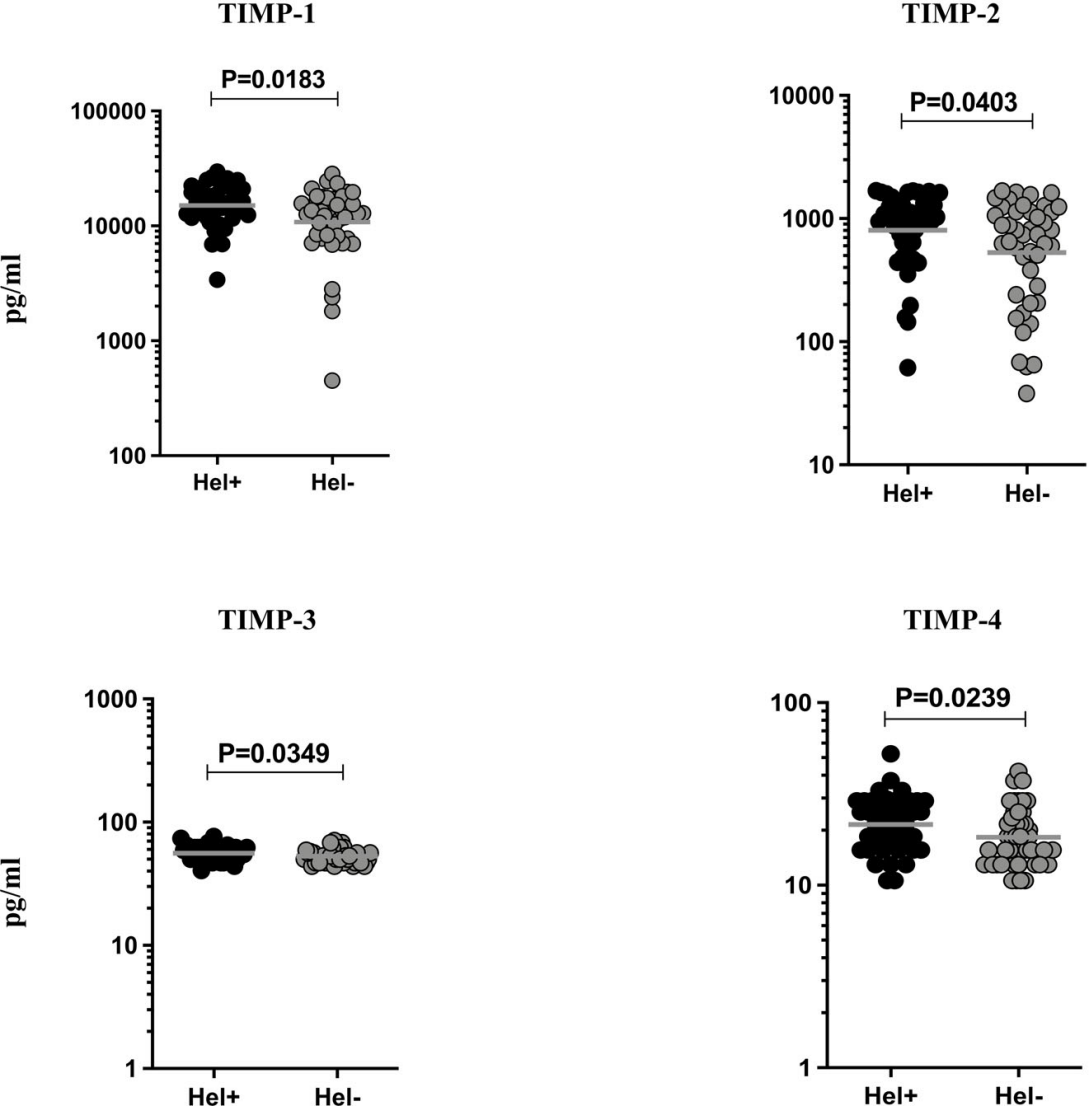
Circulating levels of tissue inhibitors of metalloproteinases (TIMPs) were elevated in TBL-Hel+ coinfected individuals. The systemic levels of TIMPs (1, 2, 3, 4) were examined in TBL-Hel+ (n = 44) and TBL-Hel- (n = 44) individuals. The results are given as scatter plots with each circle indicating a single individual and the bar representing the geometric mean. P values were calculated using Mann-Whitney U test.

To study the effect of helminth coinfection on TBL disease, we examined the plasma levels of TIMPs (1, 2, 3, 4) in TBL-Hel+ and TBL-Hel- individuals (**Figure 2**). The plasma levels of TIMP-1 (GM of Hel+ is 15072 pg/ml vs 10828 pg/ml in Hel-, p=0.0183), TIMP-2 (GM of Hel+ is 805.1 pg/ml *vs* 529.8 pg/ml in Hel-, p=0.0403), TIMP-3 (GM of Hel+ is 56.05 pg/ml *vs* 53.02 pg/ml in Hel-, p=0.0349) and TIMP-4 (GM of Hel+ us 21.54 pg/ml vs 18.30 pg/ml in Hel-, p=0.0239) were significantly elevated in TBL-Hel+ coinfected individuals compared to TBL-Hel- individuals. Thus, heightened circulating levels of TIMPs are the characteristic feature of TBL-Hel+ coinfection.

### MMP/TIMP Ratio of TBL Helminth Coinfection

**Figure 3:**
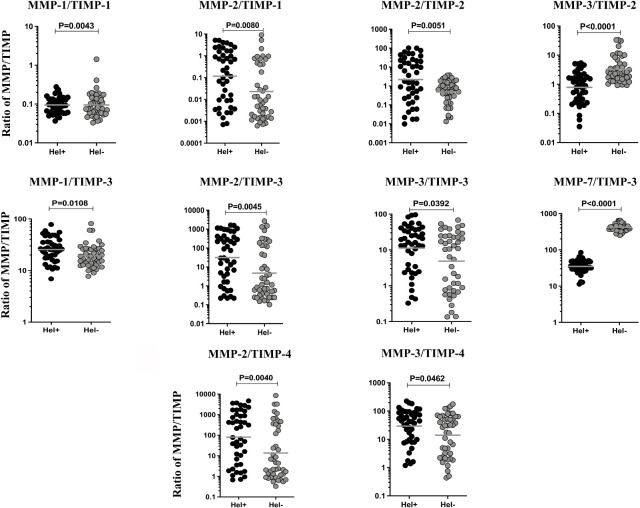
Ratio of MMP/TIMP were altered in TBL-Hel+ coinfected individuals. The ratio of different MMPs (1, 2, 3, 7, 8, 9, 12, 13) and each one of the TIMPs (1, 2, 3, 4) were compared between TBL-Hel+ (n = 44) and TBL-Hel- (n = 44) individuals. The ratios were represented as scatter plots with each circle denoting a single individual and the bar indicating the geometric mean. P values were calculated using Mann-Whitney U test.

We then examined the ratio between MMPs (1, 2, 3, 7, 8, 9, 12, 13) with each of the TIMPs (1, 2, 3, 4) in helminth coinfection with TBL disease compared to TBL-Hel- individuals. As shown in **Figure 3**, TBL-Hel+ individuals had significantly altered MMP (1, 2) with TIMP-1, MMP (2) with TIMP-2, MMP (1, 2, 3) with TIMP-3, MMP (1, 2, 3) with TIMP-4 and MMP (3, 7) with TIMP (2, 3) ratio when compared to TBL-Hel- individuals (**Figure 3**). Thus, MMP-2/TIMP (1, 2, 3, 4) ratios are elevated in TBL-helminth coinfection.

### Regression Analysis of MMPs and TIMPs

Finally, we examined the univariate and multivariate analysis (with 95% confidence interval [CI]) of MMPs (1, 2, 3, 7, 8, 9, 12, 13) and TIMPs (1, 2, 3, 4) in TBL-Hel+ and TBL-Hel- coinfected individuals upon normalizing the confounding (age, sex and culture status) factors to understand the potential ability to discriminate between the study groups. Our data reveals that MMP-1, MMP-2, and MMP-3 were correlated with the elevated risk of TBL-Hel+ disease. Likewise, among various TIMPs, TIMP-1, TIMP-3, and TIMP-4 were associated with increased risk of TBL-Hel+ disease (**Table 3**).

**Table 3:** Logistic regression model between TBL-Hel+ and TBL-Hel- individuals.

	Univariate		Multivariate	
	OR (95% CI)	P value	*aOR (95% CI)	P value
**MMP1**	2.19 (1.23–3.91)	0.008	2.32 (1.27–4.25)	**0.006**
**MMP2**	1.12 (1.03–1.21)	0.005	1.14 (1.05–1.24)	**0.003**
**MMP3**	1.21 (1.02–1.43)	0.026	1.23 (1.03–1.46)	**0.023**
**MMP7**	2.13 (0.87–5.21)	0.097	2.24 (0.89–5.65)	0.088
**MMP8**	0.99 (0.76–1.29)	0.940	0.93 (0.69–1.23)	0.596
**MMP9**	3.18 (0.64–15.69)	0.155	2.45 (0.45–13.34)	0.298
**MMP12**	1.62 (0.19–14.06)	0.659	1.67 (0.19–14.84)	0.648
**MMP13**	5.44 (0.87–34.03)	0.070	5.10 (0.74–35.41)	0.099
**TIMP1**	1.26 (0.86–1.84)	0.232	2.55 (1.20–5.43)	**0.015**
**TIMP2**	1.41 (1.01–1.96)	0.045	1.37 (0.98–1.92)	0.064
**TIMP3**	10.63 (1.01–112.35)	0.049	14.68 (1.24–173.47)	**0.033**
**TIMP4**	2.14 (0.94–4.87)	0.070	2.40 (1.02–5.66)	**0.046**

*Multivariate model was adjusted for age, sex and culture status.

Bold values indicates the significance.

## Discussion

In TB, MMP levels were increased in peripheral blood and at the site of infection and their systemic levels precisely replicated the disease pathology (Elkington et al., 2011a). In the case of parasitic infections, only a few studies examined the levels of MMPs and TIMPs (Gomez et al., 1999; Tsai et al., 2008; Verma et al., 2011; Maretti-Mira et al., 2011; Geurts et al., 2012). Helminth infections are capable of altering the expression pattern of both MMPs and TIMPs and some of them can act as an activator of these factors (Wynn, 2007; Anuradha et al., 2012). Nevertheless, no studies have explored the role of MMPs and TIMPs in TBL-helminth coinfection specifically in endemic settings for both the disease. Our results reveal that systemic levels of both MMPs (1, 2, 3, 7, 9, 13) and TIMPs (1, 2, 3, 4) were significantly heightened in TBL-Hel+ coinfected pathological situations.

Certain MMPs (1, 2, 3, 9) are associated with enhanced expression and activity upon infection with *Mycobacterium tuberculosis* (Mtb) (Sathyamoorthy et al., 2015). Previous observations also illustrate that MMPs are higher (1, 2, 3, 8, and 9) in TB infected sputum samples (Ugarte-Gil et al., 2013). Other studies have also shown that various MMPs have been enhanced in TB patients compared to controls (Hoheisel et al., 2001; Elkington et al., 2011b; Sundararajan et al., 2012; Seddon et al., 2013; Sathyamoorthy et al., 2015). The levels of MMPs (1, 2, 8, 9) are increased in pleural fluid of TB patients than non-TB pleuritis (Walker et al., 2012). MMP-9 level was significantly greater in cerebrospinal fluid (CSF) of meningeal TB which associates with their neurological compromise (Matsuura et al., 2000; Price et al., 2001). The levels of MMPs increased in TB-HIV+ coinfected sputum samples when compared to TB-HIV- controls (Walker et al., 2017). Finally, plasma levels of certain MMPs (1, 2, 3, 7, 10, 12 and 13) were significantly increased in TB-diabetes mellitus (DM) coinfected individuals than TB and HC individuals (Kumar et al., 2018). Our data were similar to previous observations and we reveal that systemic levels of MMPs (1, 2, 3, 7, 9, 13) were significantly elevated in TBL-Hel+ coinfected individuals compared to TBL-Hel- individuals. The greater secretion of various MMPs suggests TBL-helminth coinfection could induce extensive degradation of the basement membrane, proteolytic cleavage of tissue matrix, collagen breakdown, and thereby enhances the inflammatory responses in the human host. In addition, the ECM degradation could influence the dissemination of TB infection, which in turn compromises the protective immune responses against TBL infection. Up regulated MMP levels also facilitate the migration of leukocytes into the circulation. This often leads to necrosis and cavitation and thus creates an immune-privileged site for bacterial multiplication. Thus, helminth infection is likely to have detrimental effects on the pathogenesis of TBL disease.

Previous studies have also demonstrated that TIMP-1 and TIMP-2 levels are significantly higher in TB patients than in healthy individuals (Hoheisel et al., 2001; Thrailkill et al., 2009; Puthiyakunnon et al., 2014; Walker et al., 2017; Stek et al., 2018). Similarly, plasma levels of TIMP-1 and TIMP-2 were significantly increased in chronic filarial pathology patients (Anuradha et al., 2012). In contrast, TIMPs (1, 2, 4) levels were significantly diminished and TIMP-3 levels were higher in active TB-Ss+ coinfected individuals (George et al., 2014). Even in TB-Type 2 DM, comorbid patients exhibit higher plasma levels of TIMP-4 but not the other TIMPs when compared to TB disease alone (Andrade et al., 2014). Similarly, our results display that TIMPs (1, 2, 3, 4) were elevated in TBL-Hel+ infected individuals. TIMPs themselves might also be involved in the tissue destruction and hence the TBL-Hel+ infected levels exhibited enhanced TIMPs than the uninfected individuals. In contrast to TBL disease, some of the TIMP levels were reduced in pulmonary TB-helminth coinfection and this is might be because TBL is a more disseminated form than active TB, therefore, tissue destruction could also be higher in those individuals.

Finally, some of the MMPs (1, 2, 3) and TIMPs (2, 3) were linked with an elevated risk of TBL-Hel+ coinfection whereas other markers are negatively associated with enhancing the risk of TBL-helminth coinfection. In addition, TBL-Hel+ individuals had significantly elevated ratios of MMP (1, 2, 3) with all TIMPs and significantly lower ratios of MMP (3, 7) with TIMPs. The possible reason for lower MMP3/TIMP2 and MMP7/TIMP3 ratio might be because they have been reported to be mostly associated with tissue destruction and activation upon TB infection. However, the mechanism involved in the significant increase in the systemic levels of MMPs and TIMPs upon TBL-helminth coinfection requires additional study.

Our study has certain limitations from being a cross sectional study and not being able to include the helminth infected individuals for comparison. Our future studies will examine the pro-inflammatory (IFN-γ, IL-6, IL-12) and anti-inflammatory (IL-4, IL-5, IL-10, TGFβ) cytokines to understand their role in stimulating MMPs and TIMPs in the coinfected groups. Overall, our data precisely display the importance of MMPs and TIMPs in TBL-helminth coinfection and their elevated levels potentially increase the risk of disease or compromise protective immunity against TBL disease. Hence, finding the potential inhibitory molecules for MMPs and TIMPs might be important and can be considered as an adjunct therapy for treating TB and TB-helminth coinfections.

## Data Availability Statement

The original contributions presented in the study are included in the article/supplementary material. Further inquiries can be directed to the corresponding author.

## Author Contributions

Conceived and designed the experiments: GK and SB. Performed the experiments: GK, KM, and KT. Analyzed the data: GK. Contributed materials/reagents/analysis tools: DB, RS, and SB. Wrote the paper: GK and SB. All authors contributed to the article and approved the submitted version.

## Funding 

This study was supported by the Division of Intramural Research, National Institute of Allergy and Infectious Diseases (NIAID).

## Conflict of Interest

The authors declare that the research was conducted in the absence of any commercial or financial relationships that could be construed as a potential conflict of interest.
